# Raising awareness of uncertain choices in empirical data analysis: A teaching concept toward replicable research practices

**DOI:** 10.1371/journal.pcbi.1011936

**Published:** 2024-03-28

**Authors:** Maximilian M. Mandl, Sabine Hoffmann, Sebastian Bieringer, Anna E. Jacob, Marie Kraft, Simon Lemster, Anne-Laure Boulesteix

**Affiliations:** 1 Institute for Medical Information Processing, Biometry and Epidemiology, Medical Faculty, Ludwig-Maximilians-Universität München, München, Germany; 2 Munich Center for Machine Learning (MCML), München, Germany; 3 LMU Open Science Center, München, Germany; 4 Department of Statistics, Ludwig-Maximilians-Universität München, München, Germany; bioinformatics.ca, CANADA

## Abstract

Throughout their education and when reading the scientific literature, students may get the impression that there is a unique and correct analysis strategy for every data analysis task and that this analysis strategy will always yield a significant and noteworthy result. This expectation conflicts with a growing realization that there is a multiplicity of possible analysis strategies in empirical research, which will lead to overoptimism and nonreplicable research findings if it is combined with result-dependent selective reporting. Here, we argue that students are often ill-equipped for real-world data analysis tasks and unprepared for the dangers of selectively reporting the most promising results. We present a seminar course intended for advanced undergraduates and beginning graduate students of data analysis fields such as statistics, data science, or bioinformatics that aims to increase the awareness of uncertain choices in the analysis of empirical data and present ways to deal with these choices through theoretical modules and practical hands-on sessions.

## Introduction

Statistics and data analysis education frequently focuses on acquiring skills and techniques concerning specific topics that are covered successively and in isolation. Students may, for instance, first take a course on general techniques for regression modeling without considering the challenges associated with missing data, outliers, or nonrepresentative sampling mechanisms. They may then acquire skills to specifically address these additional challenges in a later course. In the classroom, students are often presented with clear examples and with clean data sets to practice these skills and techniques on. These exercises typically have unique, correct solutions to the analysis task and often yield significant results, possibly conditioning students to expect the same from real-world data. In this vein, problems arising during the analysis are considered in isolation, even though they occur simultaneously and may be interrelated. While the simplified and sequential treatment of specific topics certainly makes sense from a pedagogical standpoint, it may convey the unrealistic expectation that for any data analysis task, there is a unique and correct analysis approach that will always yield a significant or interesting finding. This expectation is further strengthened when reading published research articles in which the authors commonly describe a single analysis strategy and report a significant finding without a detailed discussion of alternative analysis options.

This impression conflicts with a growing realization that there is a multiplicity of possible analysis strategies when analyzing empirical data [1–3] and that data analysts require the ability to make subjective decisions and acknowledge the multiplicity of possible perspectives [4]. In particular, so-called multianalyst projects [5–7] show that different teams of researchers make very different choices when they are asked to answer the same research question on the same data set. These uncertain choices, which are also referred to as researcher degrees of freedom [8,9], can be combined with result-dependent selective reporting to obtain the “most noteworthy” or impressive results. This is a practice known as “p-hacking” or “fishing for significance” in the context of hypothesis testing and, more generally, “fishing expeditions” or “cherry-picking.” These practices lead to overconfident and nonreplicable research findings in the literature and, ultimately, to situations where some may argue that “most published research findings are false,” especially in combination with a low prior probability of the hypothesis being true [10,11]. Computational biology as a field is, unfortunately, not immune to these types of problems [3,12].

For example, Ullmann et al. [3] show how the combination of researchers’ expectations and selective reporting may lead to overoptimistic results in the context of unsupervised microbiome analysis. Their paper highlights the relevance of open science practices in the field of computational biology.

Here, we argue that if students always encounter clean data sets with a correct unique analysis strategy yielding a significant and/or noteworthy finding during their training, they are ill-equipped for real-world data analysis tasks and unprepared for the dangers of selectively reporting the most promising results. In particular, data analysis courses commonly teach students to understand and apply statistical models, but in order to equip them against the cherry-picking, we need to strengthen awareness and understanding of uncertainties in the analysis of empirical research data. To address this point, we present a seminar course intended for advanced undergraduates and beginning graduate students of data analysis fields such as statistics, data science, or bioinformatics that aims to increase awareness of the multiplicity of analysis strategies and of ways to deal with this multiplicity through the introduction of theoretical concepts and practical hands-on sessions.

The remainder of the article is organized as follows: Section “Teaching concept” presents the general teaching concept of the proposed seminar course. Section “Implementation and student feedback” provides evidence on the instructional value of the proposed course. Section “Potential adaptations” discusses potential adaptations of the course, and in Section “Conclusion,” we highlight key skills and takeaways that we hope students will gain.

## Teaching concept

### Overview

The course consists of theoretical modules and practical hands-on sessions. It starts with two short lectures, providing a brief introduction to the concepts of reproducibility and replicability. Subsequently, it focuses on reproducibility by introducing the students to version control software and R-Markdown to make their analyses reproducible, i.e., they learn to prepare their code in a way that all results can be reproduced “by mouse click.” In this paper, we follow the definition by Nosek et al. [13], i.e., reproducibility involves verifying the reliability of a previous discovery by employing the identical data and analysis strategy.

The second part of the course is devoted to replicability in a broad sense, where a result is said to be replicable if one obtains a similar result when repeating the same study including the collection of independent data. More specifically, the students participate in a hands-on session, in which each student is asked to perform a regression analysis on the same data set. After this first hands-on session, they are presented with a second theoretical module that focuses on uncertain choices in the analysis of empirical data, the consequences of result-dependent selective reporting, and ways to address these issues. While the hands-on session can be seen as an evaluation of the extent of selective reporting in the classroom, this second theoretical module can be seen as an intervention. It aims to prevent the students from selectively reporting the most promosing results arising through the multiplicity of possible analysis strategies. The effect of this intervention can, to some extent, be measured by comparing the results of the first phase of the hands-on session with a second phase, which follows the theoretical module on researcher degrees of freedom, in which the students are again asked to analyze a data set that has been generated according to the same model and parameter values as the data set in phase 1. The students’ experience with the two hands-on sessions, the results concerning this intervention effect, and their takeaways are discussed in the last two sessions of the course. A sample weekly schedule for a 10-week academic term is shown in Table 1. Note that the course might alternatively be conducted as an intensive course in one or few days as discussed in the section on potential adaptations.

**Table 1:** Sample weekly schedule for a 10-week academic term.

Topic	Details
Week 1–2: Reproducibility	Introduction to version control software (Git, GitLab) and R-Markdown.
Week 3: Phase 1 hands-on session	First assignment: 3-hour onsite task.
Week 4–7: Introduction of theoretical concepts	Lectures on uncertain choices in the analysis of empirical data, consequences of result-dependent reporting of analysis strategies, and ways to address these issues.
Week 8: Phase 2 hands-on session	Second assignment: 3-hour onsite task.
Week 9–10: Debriefing:	Review and discussion of results and the data generation process of the simulation setup.

### Practical hands-on sessions

In the two hands-on sessions, which should ideally take around 3 hours and be onsite to guarantee that there is no exchange between the students, each student receives the same simulated data set and is asked to estimate the effect of a predictor of interest in a linear regression model and to provide a point estimate and a 95% confidence interval. See Section C “Instructions for the students” in S1 Appendix for more details on the exact instructions received by the students.

The analysis task is designed in such a way that several uncertain choices related to model selection, treatment of missing values, and handling of outliers are required. Although we realize that such questions should ideally be tackled at the design stage of a study, in practice many researchers unfortunately address these difficulties post hoc.

To help the students with these choices, they are provided with literature that gives an overview of methods and guidance on these choices (see, for instance, [14,15]) and they are able to ask the lecturer for advice during the entire session. Additionally, the students are given information on the “likely range” of the effect of interest, while the true effect is somewhat below this range. The goal is to mimic a realistic data analysis situation in which the life scientist may hope for a large effect and exert gentle pressure on the data analyst toward observing it in the data. For each of the hands-on sessions, students are asked to analyze the data in the best possible way (which is not necessarily the same for both phases) and to hand in their results and reproducible analysis code.

### Theoretical module on uncertain choices in the analysis of empirical data and ways to address them

The theoretical module consists of lectures that address the ubiquity of uncertain choices in the analysis of empirical data, their consequences on the validity of statistical inference if they are combined with selective reporting, and solutions to address this issue. In particular, the lectures detail how result-dependent selective reporting (cherry-picking, HARKing [16], and selective publication of significant findings) can lead to overoptimism. Further, they outline that there is increasing evidence that this practice is both common and detrimental for the replicability and credibility of the scientific literature.

Finally, as an outlook, the theoretical module also presents general strategies to deal with the multiplicity of possible analysis strategies while preserving the validity of statistical inference. This can include preregistration, blind analysis, and multiverse-style analyses. A list of articles that can be used to design this theoretical module can be found in Section A “Details on the implementation” in S1 Appendix.

### Debriefing

The last two sessions leave space for the discussion of the results of the two hands-on sessions, of the students’ experience with the course, and of student takeaways. In the first session, the students are presented with the results of the first hands-on session in which they analyzed the same data set. Due to the uncertain choices in the analysis of this data set, it is likely that the students chose a variety of analysis strategies and obtained different results, providing them with first-hand experience that there is not a single correct analysis strategy for every data analysis task. These results are then compared with the true parameter value that was used to generate the data, providing insight to the extent of selective reporting that was performed during the analysis. Instructors may stress that true parameter values are not known in real data analysis and point out the principles of statistical simulations and their importance for data analysis methods by mimicking real-world scenarios with known truth.

In the second debriefing session, the results of the two hands-on sessions are compared to assess the intervention effect of the theoretical module on uncertain choices in the analysis of empirical data. As seminar courses tend to be small (with less than 30 students) and some students might lack motivation or skills to either perform multiple analyses (and selective reporting) in the first hands-on session or to change their analysis strategy in the second hands-on session, it is unlikely that a statistically significant intervention effect would be observed. Such a nonsignificant finding opens the discussion to reasons for this “failed experiment,” including lack of power, imperfect adherence and, more generally, that this nonsignificant finding cannot be interpreted as evidence that the intervention is useless since “absence of evidence is not evidence of absence” [17] and that practical importance and significance are distinct concepts [18]. After discussing the realities of experimental design, the lecturer can present the students with alternative possible results on this intervention effect resulting, for instance, from more or less plausible inclusion and exclusion criteria or outcome switching that would lead to a statistically significant intervention effect. This could raise student awareness of their own preconceived expectations that it is only a matter of finding the right analysis strategy to produce an intended result. This is a common fallacy that can arise, especially in the analysis of underpowered studies.

## Implementation and student feedback

We implemented a version of the course concept described in Section “Teaching concept” as a seminar course for advanced undergraduate students in statistics at Ludwig-Maximilians-Universität München (Germany) in 2021/2022.

The overall feedback from the students was very positive and indicated that the course had the intended effect of raising awareness of uncertain choices in the analysis of empirical data and of the dangers of result-dependent selective reporting.

The following 2 student statements, which we received after asking the students for more detailed feedback, further support this conclusion:

“I think that the learning effect of the seminar was greater than in a classical seminar, which consists exclusively of frontal teaching and presentations. […] This also made me aware of how difficult it is to make statistical decisions on the basis of the available information.”

“The seminar was very practical compared to other seminars, which made itself and the experience unique. This seminar and the experiment have had a sustainable effect on the way I do statistics. For example, it is okay to get an inconclusive result when analysing data, not everything has to be significant.”

**Fig 1:**
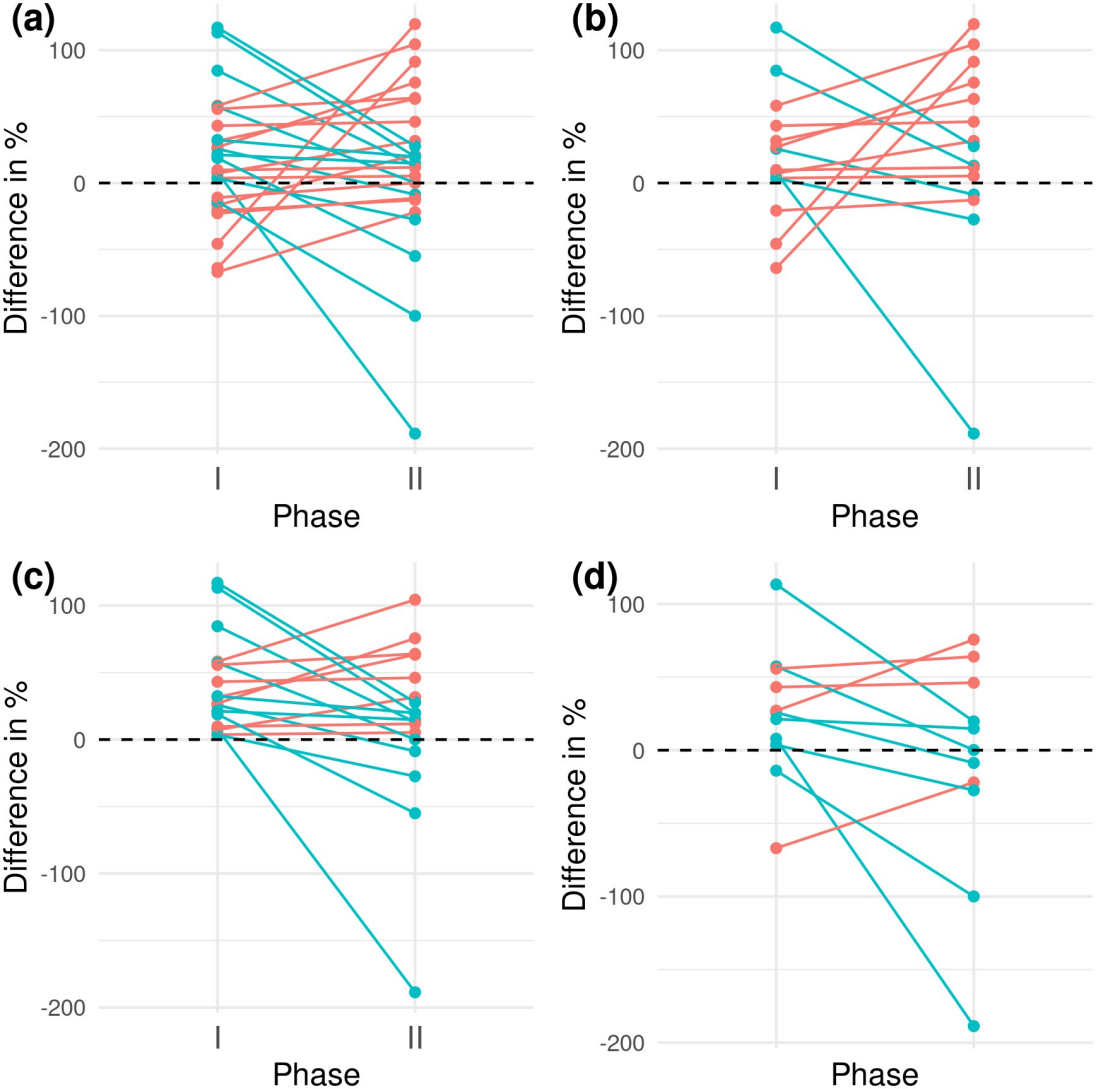
Difference in % between the estimated and true effects (represented as relative under- or overestimation) in phases 1 and 2. (a) Full sample (*n* = 26); (b) students with higher grades (*n* = 15); (c) students who overestimated the true effect in phase 1 (*n* = 18); (d) female students (*n* = 11). Connected points represent the values for phases 1 and 2 for each student. Red lines indicate an increased estimated effect size in phase 2 compared to phase 1, and blue lines indicate the reverse.

Fig 1 shows the difference between the estimated and true effects (represented as relative under- or overestimation) in phases 1 and 2 for the full sample (*n* = 26) and 3 further selected subsamples. In phase 1, the students reported a parameter estimate that was on average 17.55% larger than the true parameter value (one-sided *t* test: *p* = 0.03; Wilcoxon: *p* = 0.04), indicating that our instructions may indeed have incited the students to selectively report promising results.

In phase 2, the reported effect was on average 11.67% larger than the true effect (one-sided *t* test: *p* = 0.18; Wilcoxon: *p* = 0.05), providing less evidence for result-dependent selective reporting after the theoretical module on uncertain choices and their consequences for the validity of statistical inference. Even if there was a significant overestimation of the effect in phase 1 (17.55%) but not in phase 2 (11.67%), the 2 phases did not significantly differ with respect to this difference (paired one-sided *t* test: *p* = 0.35; Wilcoxon: *p* = 0.40), a result that may appear counterintuitive to students and is certainly worth pointing out.

An aspect worth being discussed with the students is shown in Fig 1. The intervention effect becomes significant (or very close to the 5% level) if we (slightly) change our analysis strategy, for instance, by performing the analysis only on students who overestimated the effect in phase 1 (Fig 1(c): *n* = 18, paired one-sided *t* test: *p* = 0.04; Wilcoxon: *p* = 0.06) or only on female students (Fig 1(d): *n* = 11, paired one-sided *t* test: *p* = 0.06; Wilcoxon: *p* = 0.09), leaving room for the selective reporting of promising intervention effects in this highly underpowered experiment. Conversely, the *p*-value of the intervention effect can also increase if we include only the students who performed well in terms of grades in the course (Fig 1(b): *n* = 15, paired one-sided *t* test: *p* = 0.57; Wilcoxon: *p* = 0.68).

For more details, see Sections A “Details on the implementation,” B “Data simulation,” and C “Instructions for the students” in S1 Appendix. The code and data can be found on GitHub (https://github.com/mmax-code/teaching_concept).

## Potential adaptations

Since the multiplicity of possible analysis strategies and result-dependent selective reporting are complex issues with many different aspects, there are several potential adaptations that can be made to tailor the course to varying preferences and needs.

In our implementation of the course, we chose to have the students work on simulated data sets, but it is of course possible to choose real data sets for the hands-on-sessions. To decide between these two options, it is important to decide whether one merely intends to raise awareness for the multiplicity of possible analysis strategies or to caution students against the dangers of result-dependent selective reporting. More generally, questionable research practices that may result from this multiplicity of possible analysis strategies include HARKing, fishing for significance, and data dredging. In the case where the aim is to caution against result-dependent selective reporting, it is indispensable to use simulated data sets in the hands-on session to be able to show how these practices lead to an overestimation of the true parameter value (which would be impossible on a real data set since the true parameter value is unknown). If, on the other hand, the course only focuses on raising awareness of uncertain choices and the multiplicity of possible analysis strategies, it seems more advisable to use real data sets with all their “ugly” features including, for instance, complex patterns of missing data and outliers since they offer a more realistic framework to achieve this teaching purpose, in the vein of the multiverse analysis in the classroom suggested by Heyman and Vanpaemel [19].

A second important decision in the teaching concept concerns the question of whether to focus on long-term strategies to address the multiplicity of possible analysis strategies or to present students with short-term solutions whose effects will be more observable when comparing the results from the first and the second phase of the hands-on session. The course concept that we presented here was designed to be instructive in the long term (such an effect being impossible to demonstrate in the course setting) rather than to show a large intervention effect. In this sense, the strategies that we presented to prevent result-dependent selective reporting included preregistration, blind analysis, and multiverse analyses. While these strategies are indubitably very helpful for students to address the multiplicity of possible analysis strategies in future projects, they may be of rather limited value in the second hands-on session of the course.

Related to this latter point, we chose the timing of the course to be rather early in the students’ curriculum to inoculate them against result-dependent selective reporting among a multiplicity of possible analysis strategies. This is hopefully before they were even aware of the wealth of methods and modeling strategies that they could choose from. While we believe that this may very well increase the long-term effectiveness of the teaching intervention, it will inevitably reduce the size of the intervention effect that we can observe when comparing the first and the second phase of the hands-on session because this lack of awareness reduces the number of analysis strategies that the students can choose from. In contrast, one could choose a later timing of the course in the students’ curriculum or provide the students with abundant literature on various methods and include additional lectures on methods (for instance, on model selection or missing values) in the course. In our implementation of the course of limited volume, we deliberately decided not to handle methodological issues beyond a brief introduction, in order to focus on reproducibility and replicability. The fact that students used (mostly the same) rather simple methods (for instance, AIC-based model selection) in the implementation suggests that they were probably not aware of the many possibilities they had—which may de facto prevent them from fishing for significance. Presenting the students with a multiplicity of methods before or during the hands-on sessions, on the other hand, might increase their fishing behavior, at least in the first hands-on session. Finally, we did not explicitly ask the students to change their analysis strategies, which may have led students with limited motivation to keep the same analysis strategy for both phases.

This focus on the long-term effectiveness of the course rather than on short-term strategies that may be perceivable in the comparison of the first and the second hands-on session might very well explain why we did not observe a significant reduction in result-dependent selective reporting between the two phases. However, as pointed out in Section “Debriefing,” we would consider this nonsignificant result less of a bug and more of a feature since it opens the discussion to topics including lack of power, imperfect adherence and, more generally, reminds the students that a nonsignificant finding cannot be interpreted as evidence that an intervention did not work.

On a completely different level, the course could be adapted to other types of data analyses in a broad sense beyond the generic example of effect estimation with regression models considered here. Selective reporting is relevant and may be considered in various contexts such as supervised learning [20], cluster and network analysis [3], or gene set analysis [21] rather than statistical testing in regression models. Examples inspired from these studies may be appropriate for students majoring in fields related to computational biology. Note that even though a prerequisite for our course is the use of an interpreted programming language such as R or Python and at least basic knowledge of regression models, the general concept of the course can, in principle, also be applied to students with a weaker computational background. For example, one could implement the course with a simple hypothesis test setting using a statistical software framework including a user interface (for instance, SPSS).

Finally, depending on the complexity of the considered analyses and the amount of effort required from students to understand and execute the analyses, the course concept could also be adapted to a one or multiple day intensive course. With such a shorter format, the complexity of the hands-on task and the width of the covered theoretical topics (see section A in S1 Appendix) should probably be reduced compared to our original version of the course. For example, one could address primarily the multiplicity of analysis strategies and put less focus on specific software aspects (such as the use of R-Markdown).

## Conclusion

There has been growing evidence in recent years that the current use (and misuse) of data analysis methods has contributed to what has been referred to as a “replication crisis” or “statistical crisis” in science. We argue that we need to address these problems in the way we teach statistics and data analysis [22]. In particular, we need to raise awareness regarding the potential dangers of selective reporting in the education of computational scientists. With the concept of the presented course, we address this issue through practical hands-on sessions and theoretical modules. Going beyond selective reporting, the course also provides the opportunity to teach students reproducible research practices [23] and to discuss important issues in the design and analysis of experimental studies, including lack of statistical power, nonadherence, and the common misinterpretation of absence of evidence as evidence of absence.

While the combination of a multiplicity of possible analysis strategies with selective reporting is an important issue today, it is likely to pose even more challenges in the future with the increasing availability of large complex data sets. In the analysis of these data sets, researchers are faced with even more uncertain choices than in data that are collected within simple focused experiments, as there is far less knowledge of the data generating mechanisms and control over measurement procedures. To avoid what Meng [24] calls “Big data paradoxes” in the analysis of these data sets (“the more the data, the surer we fool ourselves”), we urgently need to prepare our students for the realities of empirical data analysis by fostering their awareness and understanding of uncertain choices and ways to address these choices that preserve the validity of statistical inference.

## Supporting information

S1 Appendix Details on the implementation, data simulation, and instructions for the students. (PDF)
