# Supplementary material for: Raising awareness of uncertain choices in empirical data analysis: A teaching concept toward replicable research practices
Source: PLoS Comput Biol. 2024 Mar 28;20(3):e1011936. doi: 10.1371/journal.pcbi.1011936 (PMC10977691; doi:10.1371/journal.pcbi.1011936)
Supplement: S1 Appendix — (PDF) [file pcbi.1011936.s001.pdf]

S1 Appendix

A Details on the implementation

The course and its hands-on sessions that we present in this paper were conducted in the context of a mandatory seminar course for bachelor of science in statistics students at Ludwig-Maximilians-Universität München (Germany). Its experimental setting, i.e., the measurement of a potential intervention effect, was approved by the ethical committee of the faculty for mathematics, informatics and statistics at the Ludwig-Maximilians-Universität München, Germany (EK-MIS-2021-065). Furthermore, the students gave their informed consent to participate in the experiment.
Table A shows an overview of the analytical choices of the students for phase 1 and 2. Most students (phase 1 and 2: 81% and 69%, respectively) used a stepwise AIC approach for model selection. For outlier detection, the preferred method was to visually detect them via boxplots (phase 1 and 2: 50%) and subsequently drop or adjust them according to the 97.5% and the 2.5% quantiles. Missing values were either naively dropped or mean imputed in most cases (phase 1 and 2: 84% and 79%, respectively). The rest of the students either imputed the median, imputed a value using parametric assumptions, replaced the missings with zeros, or dropped the entire variable containing missing values. Only one student in each phase implemented lasso regression for model selection in addition to the stepwise AIC approach. A few students based their model selection on univariate selection via Pearson's correlation.
The analytical choices in phase 2 were overall quite similar to phase 1. Interestingly, some students applied p-splines for the potential non-linear effect or a train/test split approach, which was not necessary in this setting. Only one student discussed the problem of the multiplicity of possible analysis strategies. This student reported two results and decided against the AIC criterion and in favor of the smaller effect size.

Phase	Set of Problems	Model selection	Outliers	Missings	Others
Phase 1	Unbalanced category, missings, interaction	no selection	Boxplots	Drop	selectively check for quadratic terms, interactions
Phase 1	Unbalanced category, missings, interaction	Manually	-	Drop	-
Phase 1	Unbalanced category, missings, interaction	Manually, AIC	Z-score	Mean imputation	-
Phase 1	Unbalanced category, missings, interaction	Manually, Stepwise AIC	-	Drop	selectively check for quadratic terms, interactions
Phase 1	Unbalanced category, missings, interaction	Stepwise AIC	Boxplots	Drop	-
Phase 1	Unbalanced category, missings, interaction	Stepwise AIC	Boxplots, Cooks-distance	Drop, Mean imputation	log-transformation
Phase 1	Unbalanced category, missings, interaction	Stepwise AIC, BIC	Boxplots	Drop	selectively check for quadratic terms, interactions, p-spline
Phase 1	Interaction, outliers	Manually, Stepwise AIC, BIC	Boxplots	-	selectively check for quadratic terms, interactions
Phase 1	Interaction, outliers	Pearson correlation	Boxplots	-	ANOVA, train-test split
Phase 1	Interaction, outliers	Pearson correlation	Boxplots, Grubbs-test	-	log-transformation, ANOVA
Phase 1	Interaction, outliers	Stepwise AIC	-	-	selectively check for quadratic terms, interactions
Phase 1	Interaction, outliers	Stepwise AIC	Boxplots	-	-
Phase 1	Interaction, outliers	Stepwise AIC	Boxplots	-	selectively check for quadratic terms, interactions
Phase 1	Interaction, outliers	Stepwise AIC	Boxplots	-	selectively check for quadratic terms, interactions, ANOVA
Phase 1	Outliers, missings	Manually, AIC	Manually	Imputation by distribution	-
Phase 1	Outliers, missings	Stepwise AIC	-	Drop	ANOVA
Phase 1	Outliers, missings	Stepwise AIC	Boxplots	Median Imputation	log-transformation
Phase 1	Outliers, missings	Stepwise AIC, BIC	Boxplots	Drop	selectively check for quadratic terms, interactions, p-spline
Phase 1	Outliers, missings	Stepwise AIC, Lasso	Boxplots	Drop	selectively check for quadratic terms, interactions
Phase 1	Interaction, quadratic term, missings	Manually, AIC	-	Mean imputation, MICE	-
Phase 1	Interaction, quadratic term, missings	no selection	-	Drop	-
Phase 1	Interaction, quadratic term, missings	Stepwise AIC	-	Mean imputation	-
Phase 1	Interaction, quadratic term, missings	Stepwise AIC	-	Replaced by 0	-
Phase 1	Interaction, quadratic term, missings	Stepwise AIC	Manually	Drop	selectively check for quadratic terms, interactions
Phase 1	Interaction, quadratic term, missings	Stepwise AIC	Z-score	Mean imputation	selectively check for quadratic terms, interactions
Phase 1	Interaction, quadratic term, missings	Stepwise AIC, Pearson Correlation	-	Drop full variable	selectively check for quadratic terms, interactions
Phase 2	Unbalanced category, missings, interaction	no selection	Boxplots	Drop	selectively check for quadratic terms, interactions
Phase 2	Unbalanced category, missings, interaction	Manually	-	Drop	-
Phase 2	Unbalanced category, missings, interaction	Manually, AIC	Z-score	Mean imputation	-
Phase 2	Unbalanced category, missings, interaction	Lasso, Stepwise AIC	-	Drop	p-spline
Phase 2	Unbalanced category, missings, interaction	Stepwise AIC	Boxplots	Drop	-
Phase 2	Unbalanced category, missings, interaction	Stepwise AIC	Boxplots	Drop	log-transformation
Phase 2	Unbalanced category, missings, interaction	Stepwise AIC, BIC	Boxplots	Drop	selectively check for quadratic terms, interactions, p-spline
Phase 2	Interaction, outliers	Manually, Stepwise AIC, BIC	Boxplots	-	selectively check for quadratic terms, interactions, p-spline
Phase 2	Interaction, outliers	Pearson correlation	Boxplots	-	ANOVA, train-test split, mixed model
Phase 2	Interaction, outliers	Bayesian model averaging	Boxplots, Grubbs-test	-	Cross validation
Phase 2	Interaction, outliers	Stepwise AIC	-	-	selectively check for quadratic terms, interactions
Phase 2	Interaction, outliers	Stepwise AIC	Boxplots	-	p-splines
Phase 2	Interaction, outliers	Stepwise AIC	Boxplots	-	selectively check for quadratic terms, interactions
Phase 2	Interaction, outliers	Stepwise AIC	Boxplots	-	selectively check for quadratic terms, interactions, ANOVA
Phase 2	Outliers, missings	Manually, AIC	Manually	Imputation by distribution	-
Phase 2	Outliers, missings	Stepwise AIC	-	Drop	ANOVA
Phase 2	Outliers, missings	Stepwise AIC	Boxplots	Median Imputation	log-transformation
Phase 2	Outliers, missings	Stepwise AIC, BIC	Boxplots	Drop	selectively check for quadratic terms, interactions, p-spline
Phase 2	Outliers, missings	Stepwise AIC	Boxplots	Drop	-
Phase 2	Interaction, quadratic term, missings	Manually, AIC	-	Mean imputation, MICE	-
Phase 2	Interaction, quadratic term, missings	no selection	-	Drop	-
Phase 2	Interaction, quadratic term, missings	Stepwise AIC	-	Median Imputation	-
Phase 2	Interaction, quadratic term, missings	Stepwise AIC	-	Replaced by 0	-
Phase 2	Interaction, quadratic term, missings	Stepwise AIC	Manually	Drop	selectively check for quadratic terms, interactions
Phase 2	Interaction, quadratic term, missings	Hypotheses testing and p-splines	Z-score	Mean imputation	-
Phase 2	Interaction, quadratic term, missings	Stepwise AIC, Pearson Correlation	-	Drop full variable	selectively check for quadratic terms, interactions

Table A. Analytical decisions in phase 1 and 2. Empty cells (-) represent no actions taken by the students. Unbalanced category means that students were left with only few observations in some categories of the categorical variable and thus needed to recode the variable.

Table B gives an overview of articles that can be used to design the theoretical module of the course. The selected topics include, among others, the multiplicity of analysis strategies, different sources of uncertainty in the analysis of empirical data, researcher degrees of freedom, p-hacking, and HARKing.

Topic	Details	Literature
Multiplicity	Introduce the multiplicity of analysis strategies and illustrate sources of uncertainties, namely measurement, data pre-processing, parameter, model, and method uncertainty	[1–5]
Researcher degrees of freedom	Introduce the topic and show how these free analytical choices may lead to an inflated type I error rate	[6]
P-Hacking	Introduce and define p-hacking and show how p-hacking leads to a change of the distribution within the area of significance	[7,8]
Strategies against p-hacking and coping with sources of uncertainty	Adjusting for multiple comparisons and create a statistical analysis plan before the analysis or data collection takes place. Present the notion of confirmatory study and pre-specified/registered data analysis protocol. Reduce uncertainty (e.g., increase sample size), report uncertainty (e.g., vibration of effects framework), accept uncertainty (e.g. replication studies), and integrate uncertainty (e.g. Bayesian framework)	[2, 9–18]
Other literature notices and topics	Vibration of effects framework; Measuring sampling, model and measurement uncertainty; multiverse analysis; HARKing; p-values; publication bias; replication	[19–28]

Table B. Articles that can be used to design the theoretical module.

B Data simulation

All data sets were simulated from a similar data generating process (DGP) with slightly different parameter values. The data included different methodological difficulties that can be addressed in different ways, yielding so-called researcher degrees of freedom. These difficulties were: interaction effects, non-linear effects, missing values, outliers, and unbalanced classes in the categorical variables. See Table A for a detailed description. Both the effect and the methodological difficulties were the same for each student in phase 1 and 2.

For the assignment in phase 1 and 2, the students received instructions on the data and the (fictive) problem at hand (see Section C Instructions for the students).

The code for the simulation of the data sets can be found on Github (`https://github.com/mmax-code/teaching_concept`). The simulation was straightforward; the covariates were drawn from a (multivariate) normal, cauchy, uniform, t-, log-normal, beta, multinomial, and binomial distribution and the response variable was built as a linear combination of some of these covariates. The covariates that did not have an effect were also included in the data set to make the model selection more complex.

We randomly allocated each of the $n = 26$ students to one of four groups, whereby the characteristics of the data sets were the same within each group; see the code for more details. The group structure was implemented to avoid collaboration between the students. In an idealized version of the course, the students should not work on the exercises remotely, i.e., the allocation to groups can be avoided.

To analyze the potential for cherry-picking present in the data sets, we analyzed the different simulated data sets within the vibration of effects framework introduced by [19]. As an example, Fig A and B show a vibration of effects plot for a representative data set, i.e., a plot that represents the -log10 p-value against the effect estimate of interest (for X_3) obtained for different model choices, where the density of the points is coded using different colors. Yellow represents the highest and purple, the lowest density. The respective quantiles (2.5%, 50%, 97.5%) are represented by the violet dashed lines for both axes. The black lines additionally mark different levels of significance (0.001 and 0.05).

10,000 randomly sampled model combinations including the variable of interest were included. Fig A shows that the density concentrates around the true effect, $\beta_3 = 0.7$, which is depicted by the vertical red dashed line. However, many points lie between $x = 0.85$ and $x = 3.1$, indicating that it was possible to selectively report results towards the given interval $I = (0.85, 3.1)$ suggested in the instructions, which is depicted by the blue shaded area.

Fig B, on the other hand, displays the results for 10,000 randomly sampled model combinations, if missing values were naively dropped and outliers were not addressed. As can be seen from the many points with abscissa larger than 0.85, it was possible to obtain overoptimistic effect estimates in I even with a naive procedure ignoring missing values and outliers. The density of the estimates has two modes within the range of our intended interval I, both corresponding to significant results. This shows that reporting overoptimistic results could be achieved quite easily within our experimental setting.

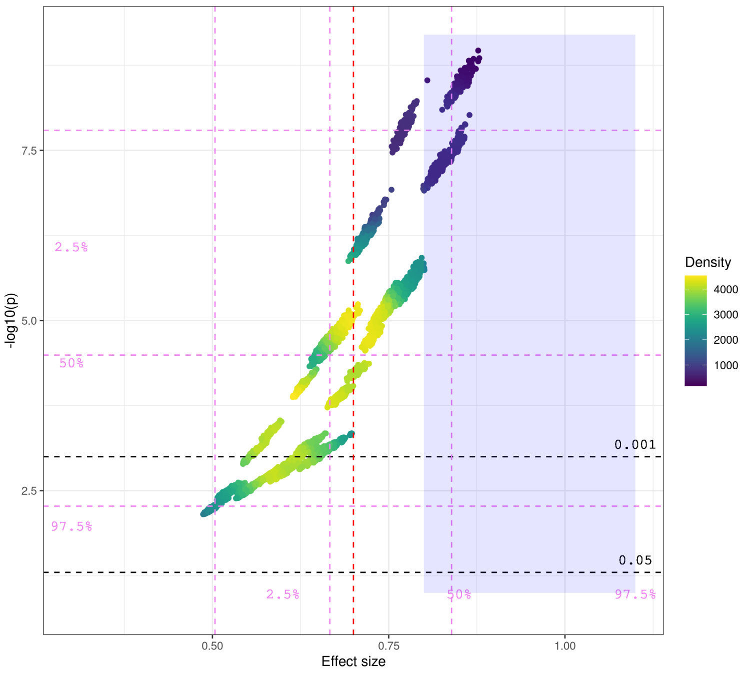

Fig A. Vibration of effects plot for 10,000 possible models (randomly sampled). Simulated data for the complete dataset without any difficulties such as interaction and non-linear effects, missing values, and outliers.

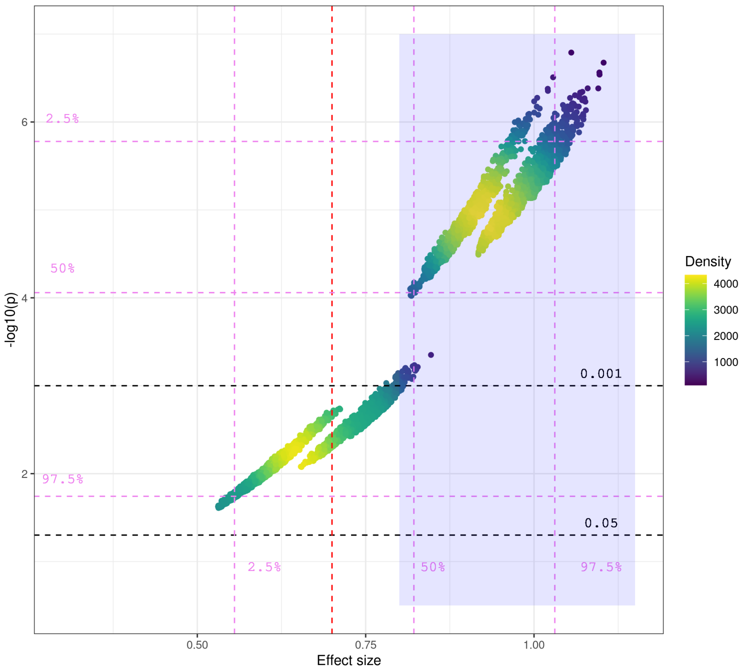

Fig B. Vibration of effects plot for 10,000 possible models (randomly sampled). Simulated data including outliers and MAR data. Missings were dropped and outliers ignored.

C Instructions for the students

Imagine yourself in the following situation[1]: You work at Ludwig-Maximilians-Universität München (Germany) and have been assigned as a statistician at Klinikum Großhadern (teaching hospital) to provide your statistical knowledge to a group of physicians. You receive a dataset with 12 variables (Y, X_1,...,X_{11}) and $n = 350$ observations. The physicians assume there is a linear effect of variable X_3 on Y, which was previously reported in scientific publications to be in the range $(0.85, 3.1)$. You also receive the following relevant information:

- X_{10} and X_{11} are categorical variables without intrinsic ordering to the categories.
- There may be further interaction effects, especially with the binary variable X_{11}. However, the literature is inconclusive on this interaction.
- Based on the variable X_6, one might suspect that the relationship is non-linear. Some studies have modeled it as non-linear, however others have modeled it linearly.
- Physicians are unsure of the effect or presence of an effect for the remaining variables.

(a) Estimate a linear regression model or related model for the situation described above. Make sure your results are reproducible, i.e., your model must always lead to the same results when you run your R-Markdown file.
(b) Explain the decisions you made during model selection and any data pre-processing procedures you followed. Typical data pre-processing procedures include, for example, handling missing values and outliers.
(c) Report the regression coefficient $\widehat{\beta}_3$ (including the confidence interval) for the variable X_3.

Note: Please avoid collaboration with classmates. Each participant has received a unique dataset, no conclusions can be drawn regarding other data sets. Your results and/or the reproducible code will be checked for similarities with your peers' work.

References

1. Steegen S, Tuerlinckx F, Gelman A, Vanpaemel W. Increasing transparency through a multiverse analysis. Perspectives on Psychological Science. 2016;11(5):702–712.
2. Hoffmann S, Schönbrodt F, Elsas R, Wilson R, Strasser U, Boulesteix AL. The multiplicity of analysis strategies jeopardizes replicability: lessons learned across disciplines. Royal Society Open Science. 2021;8(4):201925.
3. Silberzahn R, Uhlmann EL, Martin DP, Anselmi P, Aust F, Awtrey E, et al. Many analysts, one data set: Making transparent how variations in analytic choices affect results. Advances in Methods and Practices in Psychological Science. 2018;1(3):337–356.
4. Childers CP, Maggard-Gibbons M. Re: Does retrieval bag use during laparoscopic appendectomy reduce postoperative infection? Surgery. 2019;166(1):127–128.

[1]Note: The situation is fictitious and data is simulated.

5. Fields AC, Lu P, Palenzuela DL, Bleday R, Goldberg JE, Irani J, et al. Does retrieval bag use during laparoscopic appendectomy reduce postoperative infection? Surgery. 2019;165(5):953–957.

6. Simmons JP, Nelson LD, Simonsohn U. False-positive psychology: undisclosed flexibility in data collection and analysis allows presenting anything as significant. Psychological Science. 2011;22(11):1359–1366.

7. Gelman A, Loken E. The garden of forking paths: Why multiple comparisons can be a problem, even when there is no "fishing expedition" or "p-hacking" and the research hypothesis was posited ahead of time. Department of Statistics, Columbia University. 2013;.

8. Head ML, Holman L, Lanfear R, Kahn AT, Jennions MD. The extent and consequences of p-hacking in science. PLoS Biology. 2015;13(3):e1002106.

9. Wagenmakers EJ, Wetzels R, Borsboom D, van der Maas HL, Kievit RA. An agenda for purely confirmatory research. Perspectives on psychological science. 2012;7(6):632–638.

10. Klein JR, Roodman A. Blind analysis in nuclear and particle physics. Annual Review of Nuclear and Particle Science. 2005;55(1):141–163.

11. MacCoun R, Perlmutter S. Blind analysis: Hide results to seek the truth. Nature. 2015;526(7572):187–189.

12. MacCoun RJ, Perlmutter S. 15. In: Blind Analysis as a Correction for Confirmatory Bias in Physics and in Psychology. John Wiley & Sons, Ltd; 2017. p. 295–322. Available from: `https://doi.org/10.1002/9781119095910.ch15`.

13. Chambers CD. Registered reports: A new publishing initiative at Cortex. Cortex. 2013;49(3):609–610.

14. P Simmons J, D Nelson L, Simonsohn U. Pre-registration: Why and how. Journal of Consumer Psychology. 2021;31(1):151–162.

15. van 't Veer AE, Giner-Sorolla R. Pre-registration in social psychology—A discussion and suggested template. Journal of Experimental Social Psychology. 2016;67:2–12.

16. Nosek BA, Ebersole CR, DeHaven AC, Mellor DT. The preregistration revolution. Proceedings of the National Academy of Sciences. 2018;115(11):2600–2606.

17. Nosek BA, Beck ED, Campbell L, Flake JK, Hardwicke TE, Mellor DT, et al. Preregistration Is Hard, And Worthwhile. Trends in Cognitive Sciences. 2019;23(10):815–818.

18. Hardwicke TE, Wagenmakers EJ. Reducing bias, increasing transparency and calibrating confidence with preregistration. Nature Human Behaviour. 2023;7(1):15–26.

19. Patel CJ, Burford B, Ioannidis JP. Assessment of vibration of effects due to model specification can demonstrate the instability of observational associations. Journal of Clinical Epidemiology. 2015;68(9):1046–1058.

20. Klau S, Martin-Magniette ML, Boulesteix AL, Hoffmann S. Sampling uncertainty versus method uncertainty: A general framework with applications to omics biomarker selection. Biometrical Journal. 2020;62(3):670–687.

21. Klau S, Hoffmann S, Patel CJ, Ioannidis JP, Boulesteix AL. Examining the robustness of observational associations to model, measurement and sampling uncertainty with the vibration of effects framework. International Journal of Epidemiology. 2021;50(1):266–278.

22. Olsson-Collentine A, van Aert R, Bakker M, Wicherts J. Meta-analyzing the multiverse: A peek under the hood of selective reporting. PsyArXiv. 2020;.

23. Kerr NL. HARKing: Hypothesizing after the results are known. Personality and Social Psychology Review. 1998;2(3):196–217.

24. Wasserstein RL, Schirm AL, Lazar NA. Moving to a world beyond "p< 0.05". The American Statistician. 2019;73(2):129–133.

25. Turner EH, Knoepflmacher D, Shapley L. Publication bias in antipsychotic trials: an analysis of efficacy comparing the published literature to the US Food and Drug Administration database. PLoS Medicine. 2012;9(3):e1001189.

26. Nosek BA, Errington TM. What is replication? PLoS Biology. 2020;18(3):e3000691.

27. Nosek BA, Hardwicke TE, Moshontz H, Allard A, Corker KS, Dreber A, et al. Replicability, robustness, and reproducibility in psychological science. Annual review of psychology. 2022;73:719–748.

28. Goodman SN, Fanelli D, Ioannidis JP. What does research reproducibility mean? Science translational medicine. 2016;8(341):341ps12–341ps12.
